# Population Genetics of Duplicated Alternatively Spliced Exons of the *Dscam* Gene in *Daphnia* and *Drosophila*

**DOI:** 10.1371/journal.pone.0027947

**Published:** 2011-12-12

**Authors:** Daniela Brites, Francisco Encinas-Viso, Dieter Ebert, Louis Du Pasquier, Christoph R. Haag

**Affiliations:** 1 Zoologisches Institut, Evolutionsbiologie, University of Basel, Basel, Switzerland; 2 Community and Conservation Ecology Group, University of Groningen, Haren, The Netherlands; 3 Department of Biology, Ecology and Evolution, University of Fribourg, Fribourg, Switzerland; French National Centre for Scientific Research - Université Aix-Marseille, France

## Abstract

In insects and crustaceans, the Down syndrome cell adhesion molecule (Dscam) occurs in many different isoforms. These are produced by mutually exclusive alternative splicing of dozens of tandem duplicated exons coding for parts or whole immunoglobulin (Ig) domains of the Dscam protein. This diversity plays a role in the development of the nervous system and also in the immune system. Structural analysis of the protein suggested candidate epitopes where binding to pathogens could occur. These epitopes are coded by regions of the duplicated exons and are therefore diverse within individuals. Here we apply molecular population genetics and molecular evolution analyses using *Daphnia magna* and several *Drosophila* species to investigate the potential role of natural selection in the divergence between orthologs of these duplicated exons among species, as well as between paralogous exons within species. We found no evidence for a role of positive selection in the divergence of these paralogous exons. However, the power of this test was low, and the fact that no signs of gene conversion between paralogous exons were found suggests that paralog diversity may nonetheless be maintained by selection. The analysis of orthologous exons in *Drosophila* and in *Daphnia* revealed an excess of non-synonymous polymorphisms in the epitopes putatively involved in pathogen binding. This may be a sign of balancing selection. Indeed, in *Dr. melanogaster* the same derived non-synonymous alleles segregate in several populations around the world. Yet other hallmarks of balancing selection were not found. Hence, we cannot rule out that the excess of non-synonymous polymorphisms is caused by segregating slightly deleterious alleles, thus potentially indicating reduced selective constraints in the putative pathogen binding epitopes of Dscam.

## Introduction

**Figure 1:**
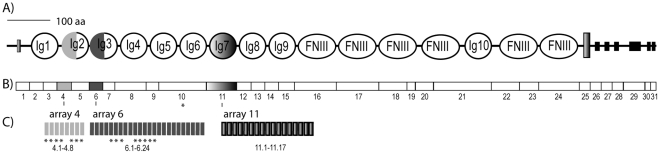
Dscam of Daphnia magna. A) Protein domains; Ig-immunoglobulin domains; FNIII- fibronectin III domains. The grey and black boxes represent the transmembrane and cytoplasmic domains. B) mRNA, each box corresponds to a constitutive exon and the colored boxes 4, 6 and 11, correspond to exons that are the result of mutual exclusive alternative splicing of arrays of duplicated exons, as indicated in C) * Dscam exons of *Daphnia* sampled in the present study.

The gene encoding Down syndrome cell adhesion molecules (Dscam) has been studied in several metazoans. It codes for an integral membrane protein with signaling capacity, the extracellular part of which is formed by immunoglobulin (Ig) and fibronectin III (FNIII) domains. In insects and crustaceans *Dscam* evolved dozens of internal exon duplications which occur in three arrays (named arrays 4, 6, and 11 in *Daphnia* and 4, 6 and 9 in *Drosophila*) [1], [2], [3]. Due to a process of mutually exclusive alternative splicing, only one exon from each array is present in each mRNA molecule. This generates thousands of mRNA molecules coding for protein isoforms that differ in half of Ig2 (coded by any exon of array 4), half of Ig3 (coded by any exon of array 6), and in all of Ig7 (coded by any exon of array 11), while keeping the remaining domains constant (Fig. 1).

In insects and crustaceans, the Dscam protein is believed to have a dual function acting both in the nervous system and in the immune system [1], [2], [3], [4]. Its involvement in the nervous system development is well established in *Drosophila* where the different protein isoforms are essential for correct axon wiring [5], [6]. The alternative splicing mechanism might be equally important for the immune function of Dscam: a diverse repertoire of Dscam isoforms is expressed in hemocytes, the immune cells of insects and crustaceans, and these isoforms can bind different bacteria depending on exon composition [1], [7]. Furthermore, the splicing patterns of the alternative exons change upon infection, and silencing of Dscam leads to lower phagocytosis rates in *Drosophila* and *Anopheles* [1], [4]. However, Dscam does not seem to be required for *E. coli* phagocytosis in *Drosophila* embryos [8]. Given that the hemocytes of adult flies are of embryonic origin these results are somewhat controversial. On the other hand, the partial blockage of bacteria uptake [1] suggests that phagocytosis is not under the control of a single pathway and it is possible that DSCAM-silenced individuals [1] behave differently from *dscam05518* mutant embryos [8] where a surrogate mechanism may take over.

**Figure 2:**
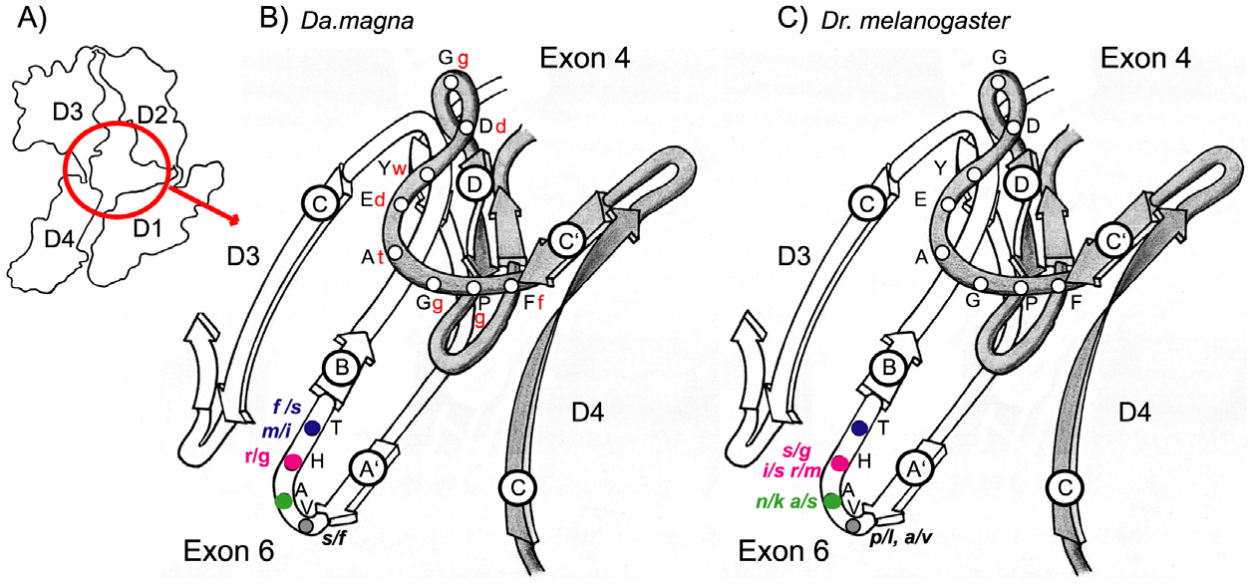
Dscam horse-shoe structure outline and detailed epitope II. A) Outline of the Dscam horse-shoe structure formed by the first four Ig domains (D1–D4). B & C) Detail of Epitope II, formed by the two interstrand loops C′-D of exon 4 and A′-B of exon 6, respectively. Each strand is indicated by an encircled letter. The *Drosophila* aminoacid residues corresponding to the actual structures are in black uppercase initials (exon 4.1 and 6.34 of *Dr. melanogaster*). *Da. magna* residues have been positioned in function of the known homology of the molecule in the region coded by exon 4 and 6 (BRITES *et al.* 2008) and are represented by red lowercase initials. Polymorphic sites at exons 6 for *Da. magna* and *Dr. melanogaster* are represented by lowercase initials, each color corresponds to positions on Epitope II coding regions in different paralogous exons 6.

The first four Ig domains of the Dscam protein form a stable horse-shoe structure, which is probably common to all isoforms [9], Fig. 2A). Parts of Ig2 and Ig3 together form two surface epitopes at either side of the horse-shoe structure, epitope I and epitope II. Both epitopes are partly coded by array 4 and partly by array 6 (Fig. 2B, Fig. S1). Epitope I is crucial for the formation of Dscam dimers and for the development of the nervous system [9]. Epitope II is oriented towards the external environment of the Dscam molecule, and is thus a candidate epitope for the interaction with antigens.

The sequence of each exon belonging to arrays 4 and 6 can be divided into parts of the sequence that contribute to epitope I, parts that contribute to epitope II, and parts that contribute to neither of them. Orthologous exons of arrays 4 and 6 show more divergence between closely related *Drosophila* species in the parts coding for epitope II than in the parts coding for epitope I [9]. This pattern, in combination with the structural features described above, has led to the idea that epitope II might be involved in host-parasite coevolution and might have evolved faster as a consequence of being a potential pathogen recognition epitope [9]. Here we address this hypothesis by searching for signatures of adaptive evolution in the nucleotide sequence coding for epitope II. We do this by analyzing polymorphism patterns of the Dscam gene in *Daphnia magna* and *Drosophila melanogaster* as well as divergence patterns between these species and some of their closely related congeners and by using molecular tests of selection, including maximum likelihood (ML) models of codon evolution.

## Materials and Methods

### Origin of the samples

We used 17 genotypes of *Da. magna*, each isolated from a different population, as well as one genotype from two outgroup species, *Da. lumholtzi* (Zimbabwe) and *Da. similis* (Israel) (Table 1). The genotypes were maintained by clonal propagation of offspring from single females isolated from these populations.

**Table 1:** Geographic origin of the Da. magna populations sampled.

Genotype	Geographic origin	Latitude	Longitude
*FA*	Tvärminne, Finland	59°50.18′N	23°14.16′E
*K-10-1*	Tvärminne, Finland	59°49.43′N	23°15.15′E
*SP1-2-3*	Tvärminne, Finland	59°48.42′N	23°12.31′E
*FAV-1-1* 1	Åland Islands, Finland	60°01.30′N	19°54.15′E
*HO1* 1	Hungary	46°48′N	19°08′E
*HO2*	Hungary	46°48′N	19°08′E
*HO3* 1	Hungary	46°48′N	19°08′E
*DKN-1-8*	Kniphagen, Germany	54°10.45′N	10°47.3′E
*MU10*	Munich, Germany	48°12.23′N	11°42.34′E
*MU11*	Munich, Germany	48°12.23′N	11°42.34′E
*GE-1*	Ismaning, Germany	48°12.23′N	11°42.34′E
*SC1*	Leitholm, UK	55°43.9′N	02°20.43′W
*EC-1-4*	Cummor, UK	51°43.9′N	01°20.4′W
*CN-2-1*	Sedlec, Czech Republic	48°46.52′N	16°43.41′E
*BE-OM-1*	Leuven, Belgium	50°52′N	04°41′E
*KE-1*	Kenia	0°26.25′N	35°18.16′E
*SE-2-3*	Sweden, East coast	60°25.93′N	18°31.34′E

1 Genotypes for which only array 6 exons were amplified, and which were only used in parts of the analysis.

The polymorphism data for *Dr. melanogaster* were obtained by [10] and come from six populations (four individuals per population pooled before DNA extraction), covering the initial range of the species in Africa and more recent expansions. The divergence data for *Drosophila* are from the sequenced genomes of six species of the *melanogaster* group obtained from gene bank (*Dr. ananassae* GF12235; *Dr. melanogaster* CG17800; *Dr. erecta* GE24114; *Dr. simulans* FBgn0086259; *Dr. yacuba* GE24114; *Dr. sechellia* CH480816). *Daphnia pulex* and other *Drosophila* species were not considered for the analysis because their synonymous site divergence was too high to allow a meaningful analysis of substitution rates due to the high likelihood of multiple hits. However, the following six additional species were included in analyses of exon copy number and analyses based on amino acid sequences only (where multiple hits are much less likely than at synonymous sites): *Dr. pseudoobscura* (GA14672), *Dr. persimilis* (CH479181), *Dr. willistoni* (CH963849), *Dr. mojavensis* (GI20826), *Dr. virilis* (GJ20560), *Dr. grimshawi* (CH916367).

### Genomic region analyzed

In *Da. magna* the entire Dscam protein, depending on exon usage, is composed of approximately 1960 amino acids and the whole locus is 31 Kb long [3]. For the present study, we analyzed three regions of the Dscam gene: two regions containing alternatively spliced, duplicated exons belonging to arrays 4 and arrays 6 (and, for comparison, one region containing the constitutive exon 10, which was chosen because it codes for Ig6, which is structurally similar to the Igs 2 and 3, coded for by arrays 4 and 6 (data not shown).

In *Da. magna*, array 4 consists of eight paralogous exons, (named 4.1 to 4.8, covering around 3390 bp in total) and array 6 contains 24 paralogous exons (6.1 to 6.24, around 6100 bp in total). We obtained sequence data on all exons of array 4, except exon 4.5 (3200 bp in total, accession numbers JN977549 to JN977579)), exons 6.5 to 6.7 and 6.10 to 6.14 (1683 bp in total, accession numbers JQ037914 to JQ037973), and 327 bp of the constitutive exon 10 (the total length of which is 423 bp, accession numbers JQ037974 to JQ037993). Part of the intron sequences (mostly from array 4) had to be excluded from the analysis due to alignment ambiguities, repetitive sequences, and insertion/deletion polymorphisms. Thus, only 1759 bp of array 4 sequences and 1679 bp of array 6 sequences were retained for analysis (Table 2). All exons sampled are known to be expressed [3]. The same sequence data was also obtained for one genotype of *Da. lumholtzi*. We were unable to obtain array 6 sequence from *Da. similis*, thus we restrict the analysis of between-species divergence mostly to divergence between *Da. magna* and *Da. lumholtzi* which is the closest known species to *Da. magna*

**Table 2:** Number of sites and number of polymorphic sites per Dscam genomic region analyzed in Da. magna (Dmag) and Dr. melanogaster (Dmel), the latter obtained from [10].

Gene region	N of sites (L)					N of polymorphic sites (S)				
	Dmag			Dmel		Dmag			Dmel	
	L_s_	L_a_	L_nc_	L_s_	L_a_	S_s_	S_a_	S_nc_	S_s_	S_a_
Array 4 total	218	731	778	458	1524	4	6	20	11	9
Epitopes I	34	117	n.a.	n.a.	n.a.	0	0	n.a.	n.a.	n.a.
Epitopes II	56	187	n.a.	120	447	2	1	n.a.	2	4
Remaining	128	427	n.a.	338	1077	2	5	n.a.	9	5
Array 6 total	213	628	728	1443	4325	17	10	27	60	46
Epitopes I	44	124	n.a.	n.a.	n.a.	1	1	n.a.	n.a.	n.a.
Epitopes II	40	128	n.a.	278	864	0	5	n.a.	29	17
Remaining	129	376	na	1164	3461	16	4	na	77	29
Ig6 coding exon	81	246	0	60	173	6	4	0	25	0

Insects have three other Dscam paralogs that have been named Dscam-like (Dscam-L) [3], [11], [12] and we have found orthologues of these *Dscam-L* genes in the genome of *Daphnia pulex* (unpublished data). The distinction between the variable *Dscam* and the *Dscam-L* genes is very clear and we are confident that we have amplified only the variable Dscam in *Daphnia*.

The Dscam sequence data from *Dr. melanogaster* [10] comprises almost the entire Dscam coding region (22795 bp). For the interspecific comparisons of the six *Drosophila* species from the melanogaster group, we used all orthologous exons of arrays 4 (12 exons, 1950 bp in total). For array 6, 43 orthologous exons were used, 32 occurring in all six species and eleven in five of them (5205 bp in total). Exons that confidently (>60% of 100 bootstrap replicates) shared a common ancestor in a maximum likelihood tree were considered orthologous [13]. Trees were built with RAxML trough the Cipres Portal [14].

### Sequencing methods

Genomic DNA of *Daphnia* genotypes was extracted (peqGOLD Tissue DNA Mini Kit, PEQLAB, Erlangen, Switzerland) and PCR reactions were carried out using High Fidelity Polymerase (ROCHE, Manheim, Germany) for array 4 exons or Pfu (PROMEGA, Madison, WI, USA) for array 6 exons and exon 10. Primers and PCR conditions are available by request. PCR products were purified (Gen Elute™ PCR Clean-up kit, SIGMA, St Louis, MO, USA), and all reactions were sequenced directly using Sanger sequencing. In addition, products of some PCR reactions were cloned (TOPO Kit, INVITROGEN, Carlsbad, CA, USA) to obtain experimental haplotype information. All heterozygous sites and singleton polymorphisms were confirmed by resequencing independent PCR reactions or cloning. To verify that only the targeted regions were amplified, all sequences were compared to a reference *Dscam* sequence, obtained by cloning the entire locus in *Da. magna* [3]. The *Dscam* sequence data from *Dr. melanogaster* was obtained by Solexa-Illumina sequencing [10]. Regions with less than 20× coverage were excluded. By resequencing eleven genes using Sanger sequencing, the authors uncovered 31 miscalled polymorphic sites in a total of 12451 bp (accuracy = 99.8%), of which 10 polymorphisms (0.08%) corresponded to false positive polymorphisms and the remaining to false negatives (0.12%) [10]. To minimize the occurrence of false positives all variants with a frequency of less than 5% within a population were excluded from the analysis [10]. Because read frequencies did not provide a reliable estimate of allele frequencies [10], the data were only used to estimate nucleotide diversity from the proportion of segregating sites (θ) and for performing McDonald-Kreitman tests [36], but not for tests based on allele frequencies.

### Identification of epitope I and epitope II coding sequences

Some analyses required partition of array 4 and array 6 exon sequences in regions that constitute epitope I, epitope II, and the remaining exon regions. These partitions were based on the structural information provided by [9] and on the similarities in the secondary structure of Dscam between *Da. magna* and *Drosophila melanogaster* (data not shown), using the program PSIPRED (http://bioinf.cs.ucl.ac.uk/psipred/) [15]. The partitions were assigned in the following way: In exons of array 4, the ten amino acids between the conserved 4Q and the 15V were considered to belong to epitope I, and the 13 amino acids after 40W were considered to belong to epitope II. In exons of array 6, the eight amino acids after 10R were considered to belong to epitope I, and the eight amino acids before the conserved LLC motive were considered to belong to epitope II (Fig. S1). Figure 2 was redrawn manually from [9] using the Dscam reference (2v5m) in the protein data bank (PDB, http://www.rcsb.org/pdb/home/home.do).

### Analysis

Sequences were assembled and edited using STADEN version 1.5 (http://staden.sourceforge.net/), aligned with ClustalX [16] and edited in Jalview 2.3 [17]. For exons of array 6, alignments including unphased sequences (7 genotypes) and true haplotypes (20 cloned haplotypes) were used to obtain pseudohaplotypes for unphased sequences using the program PHASE 2.1 [18]. For array 4 exons all PCR products were cloned. The program GENECONV version 1.81a (using default parameters) was used to detect gene conversion between paralogous exons [19].

Analyses of nucleotide diversity (π), divergence, and standard neutrality tests were done with DNAsp v5 [20]. Unless stated otherwise, divergence always refers to divergence of orthologous sequence between species, rather than divergence of paralogous sequence within species. Amino acid divergence between paralogous exons was calculated using the Poisson correction method to account for multiple substitutions at the same site, averaging over all paralogous pairs MEGA 4.0, [21].

Next, we used the site models implemented in PAML version 4 [22], [23] and HYPHY [24], [25] to test for positive selection between orthologous exons using six *Drosophila* species from the *melanogaster* group. The same models were not applied to *Da. magna* because they require data from several, closely related species. These methods assess the ratio of non-synonymous to synonymous substitutions ω = dN/dS, where ω<1 indicates purifying selection, ω = 1 neutrality, and ω>1 positive selection. They infer positive selection by asking whether a model that allows some codons to have ω>1 fits the data significantly better than a model that restricts all codons to have ω≤1.

The ML analysis was carried out in the following way: In PAML, we calculated likelihoods for the following models: M1a (assuming that sites have either 0<ωo<1 or ω1 = 1), M2a (which adds an additional class of sites with ω2>1), M7 (which uses a ß-distribution to model ω and does not allow for ω>1), and M8 (which adds an extra class of sites with ω>1 to M7). We compared the log-likelihoods between models M2a and M1a and between M8 and M7 to test for positive selection [23]. In all models, base frequencies were calculated from the average nucleotide frequencies at the three codon positions and we used the GY model [26] as basic model of codon substitution. Finally, we used the empirical Bayes approach implemented in PAML to identify individual codons under positive selection.

To account for potential differences in synonymous rates, which can influence the accuracy of detecting positively selected sites, we fitted the “dual” model implemented in HYPHY to our data [25]. We used a general discrete distribution (GDD) with three bins for dN and dS and the codon substitution model MG94 [26] combined with the nucleotide substitution model HKY85 (determined as the best-fitting nucleotide substitution model using the model selection procedure implemented in HYPHY). To identify sites under selection we used a Bayes factor of 50.

To test whether the dN/dS of epitope II regions differed from remaining of exon regions (for a similar analysis see [27] [28], we applied the ML-based hypothesis testing procedure implemented in HYPHY on two partitions of the data, one containing epitope II sequence and one containing the remaining sequence of the exons. The same tree topology and the MG94 codon model combined with HK85 nucleotide substitution model were assigned to each partition (epitope II and non-epitope II sequence) considering the observed nucleotide frequencies. For testing the hypothesis that dN/dS differs between partitions, dN/dS was estimated independently for each of them but the same tree was assumed.

To investigate substitutions patterns of paralogous exons, we applied branch models [29], [30] as implemented in PAML. This analysis was performed only on the phylogeny of exons of array 6 in the *Dr. melanogaster* group (Fig. S3A). Paralogous exons 4 have diverged too much for a reliable analysis (data not shown).Whereas orthologous exons 6 are very conserved (except epitope II coding regions), paralogous exons diverged extensively pointing out to an acceleration of aminoacid substitutions following exon duplication. Using the branch models on trees that included orthologous as well as paralogous sequences, allowed us to test whether selection changed after duplication by contrasting branches giving rise to paralogs with branches giving rise to orthologs. We used an alternative model assuming that orthologous branches and paralogous branches differ in ω (model R2, Fig. S3A and S3B), the null hypotheses being that all branches in the tree have the same ω (model R1, Fig. S3A and S3B). Under these models, ω estimates correspond to an average over branches and sites and thus unlikely to be higher than 1. We used the branch-site models implemented in PAML to test for positive selection, i.e. to test whether particular branches have aminoacid sites that evolved with a ω>1 [31], [32]. Because we did not have *a priori* data on particular exons with functional importance we chose to test the branches leading to duplicated exons where we detected an excess of non-synonymous polymorphism in *Dr. melanogaster* using MK-tests in the previous analysis. For doing this, smaller subtrees were used (Fig. S3A).

## Results

### Gene conversion and copy number of array 4 and array 6 exons

The duplicated exons of are 160 bp in array 4 and 130 bp in array 6, and within each array, they are separated by introns of approximately 200 bp (array 4) and 100 bp (array 6). None of our PCRs showed evidence (length polymorphism or failed PCRs) for variation in the number of exons in array 4, nor in array 6 (only eight contiguous exons out of 24 were investigated in the latter). We found no variation among closely related species in the number of paralogous exons in array 4: all twelve *Drosophila* species have twelve exons whereas both *Da. magna* (EU307883) and *Da. pulex* (EU307884) have eight. In contrast, array 6 has between 41 and 52 exons in the twelve *Drosophila*, and two more exons in *Da. pulex* than in *Da. magn*a. Furthermore, in *Da. lumholtzi*, at least one of the eight sampled exons of array 6 is probably missing (as indicated by our failure to obtain this sequence). This indicates that exon copy number in array 6, but not in array 4, varies among related species.

Multigene families are frequently under the action of concerted evolution by gene conversion [33]. However, consistent with earlier results based on trees of the duplicated regions in *Da. magna* and *Da. pulex* [3], we found no evidence for gene conversion between duplicated exons in arrays 4 and 6 (*p-*values based on 10000 permutations were 0.2 for array 4 and 0.5 for array 6). The low levels of polymorphism in array 4 (Table 3) may suggest gene conversion, but the high level of divergence between paralogous exons (Table 3) contradicts this hypothesis. The apparent absence of gene conversion suggests that Dscam is unusual in this respect compared with other multi-gene families and greatly facilitates further analysis because it legitimates the use of classical population genetic methods.

**Table 3:** Estimates of Dscam nucleotide diversity (π in Da magna, θ in Dr melanogaster), divergence of orthologous sequences between Da. magna and Da. lumholtzi, and amino acid divergence between paralogous regions of Da. magna, as well as divergence of orthologous sequences between Dr. melanogaster and a reconstructed ancestral sequence estimated in [10].

Species	Gene region	Diversity (π, θ)				Divergence (k)^2^				
*Dmag*	Array 4 Total	0.0014	0.004	0.005	0.0008	0.2	0.132	0.013	0.098	0.837
	Epitopes I	0	n.a.	0	0	n.a.	0.118	0.000	0	0.980
	Epitopes II	0.0014	n.a.	0.005	0.0009	0.18	0.164	0.032	0.195	1.431
	Remaining	0.0014	n.a.	0.005	0.0004	0.08	0.137	0.004	0.029	0.567
	Array6 Total	0.0064	0.01	0.017	0.003	0.176	0.148	0.013	0.088	0.593
	Epitopes I	0.003	n.a	0.003	0.0006	0.1	0.139	0.008	0.057	1.379
	Epitopes II	0.007	n.a.	0.000	0.009	n.a.	0.178	0.031	0.174	1.616
	Remaining	0.007	n.a.	0.023	0.001	0.04	0.144	0.004	0.028	0.211
	Exon10 (Ig6)	0.006	n.a.	0.011	0.005	0.454	0.149	0.003	0.02	n.a.
*Dmel^6^*	Array 4 Total	0.01	n.a.	0.024	0.006	0.25	0.039	0.003	0.077	n.a.
	Epitopes II	0.0106	n.a.	0.017	0.009	0.53	0.033	0.005	0.151	n.a.
	Array 6 Total	0.018	n.a.	0.042	0.011	0.26	0.076	0.008	0.105	n.a.
	Epitopes II	0.0253	n.a.	0.043	0.006	0.14	0.082	0.01	0.121	n.a.
	Exon7 (Ig6)	0.008	n.a.	0.033	0	n.a.	0.083	0	n.a.	n.a.
	Remaining Dscam^4^	0.019	n.a.	0.048	0.009	0.18	0.067	0.005	0.075	n.a.
	Control genes^5^	n.a.	n.a.	0.015	0.002	0.13	n.a.	n.a.	n.a.	n.a.
	Immune genes^5^	n.a.	n.a.	0.016	0.009	0.56	n.a.	n.a.	n.a.	n.a.

### General patterns of polymorphism and divergence

In *Da. magna*, array 4 has low nucleotide diversity (π) both at non-synonymous and at synonymous sites, whereas array 6 and exon 10 have moderate levels of synonymous diversity (π_s_) (Table 3), similar to the average values estimated for eight housekeeping *Da. magna* genes in another study [34], and higher than in a sample of putative immunity genes in this species [35]. In contrast, non-synonymous diversity (π_a_) in array 6 and exon 10 is about ten times higher than in other *Da. magna* genes [34]. Synonymous divergence (k_s_) between *Da. magna* and *Da. lumholtzi* is similar in all sampled Dscam regions. Contrastingly, non-synonymous divergence (k_a_) is much higher in arrays 4 and 6 than in exon 10, and correspondingly also ka/ks ratios are higher in arrays 4 and 6 than in exon 10 (Table 3). The opposite is true for the ratio of non-synonymous to synonymous nucleotide diversity ratio (π_a/_π_s_, Table 3). The divergence estimates between *Da. magna* and the second outgroup species, *Da similis* are similar to the estimates between *Da. magna* and *Da. lumholtzi*. Thus, they are presented in the supplementary materials only (Table S5) and will not be discussed further. A McDonald and Kreitman (MK)-test [36] yielded evidence for an excess of non-synonymous polymorphism compared to the ratio between non-synonymous and synonymous divergence in array 4, whereas results for array 6 and exon 10 did not differ from neutral expectations (Table 4). This is consistent with the action of balancing selection in array 4, but a Hudson-Kreitman-Aguadé (HKA) test [37] did not yield evidence for a significantly higher polymorphism to divergence ratio in array 4 compared to array 6 and exon 10 combined (synonymous sites only, p = 0.08). All non-synonymous polymorphisms in array 4 segregate at low frequencies (Table S1), so that the excess of non-synonymous polymorphism could also reflect slightly deleterious mutations. In such cases it has been suggested that removing. alleles with a frequency lower than 0.15 from the MK analysis could partially reduced the bias introduced by low-frequency polymorphisms [38]. When applying this to our data, only exon 10 has a significant excess of non-synonymous polymorphism.

**Table 4:** MacDonald Kreitman tests for the comparison between *Da. magna* and *Da. lumholtzi*.

Gene region	Raw values					Corrected MAF				
	Fixed		Polymorphic		*p* 1	Fixed		Polymorphic		*p* 1
	Syn	Nonsyn	Syn	Nonsyn		Syn	Nonsyn	Syn	Nonsyn	
Array 4 Total	28	9	4	6	0.05	28	9	1	0	1
Epitopes II	10	7	2	2	1	10	7	0	0	n.a.
Array 6 Total	26	7	17	10	0.25	29	7	4	2	0.6
Epitopes II	6	4	0	5	0.04	6	4	0	2	0.4
Exon 10 (Ig6)	10	0	6	4	0.08	12	0	0	2	0.01

1 *p* values are according to a two-tailed Fisher's exact test. n.a., not assessed.

The test was performed on raw frequencies of alleles as well on frequencies after correcting for minor allele frequency (MAF). This correction was done by eliminating all allele frequencies lower than 0.15 when considering all *Da. magna* populations.

In *Dr. melanogaster*, non-synonymous diversity is similar to that of other genes with immunity-related functions, and synonymous diversity is higher than that of other immune and control genes [10] (Table 3). In contrast to *Da. magna*, constitutively expressed and alternatively spliced exons exhibited similar levels of synonymous and non-synonymous diversity. A MK-test applied to arrays of exons 4 and 6 revealed an excess of non-synonymous polymorphism in relation to what would be expected from the divergence levels between *Dr. melanogaster* and an inferred ancestral sequence [10]. After eliminating all alleles that occurred with minor frequencies (less than 0.15) there was no longer an indication of a significant excess of non-synonymous polymorphisms in relation to divergence (Table 5).

**Table 5:** MacDonald Kreitman tests for the comparison between *Dr. melanogaster* and an ancestral sequence inferred by [10].

Gene region	Raw values					Corrected MAF				
	Fixed		Polymorphic		*p*	Fixed		Polymorphic		*p* 1
	Syn	Nonsyn	Syn	Nonsyn		Syn	Nonsyn	Syn	Nonsyn	
Array 4 Total	13	0	11	9	0.005	13	0	5	0	n.a
Epitopes II	3	0	2	4	0.16	3	0	0	0	n.a
Array 6 Total	81	14	60	46	<0.001	86	18	18	8	0.1
Epitopes II	17	7	12	17	0.051	19	7	2	7	0.01
Exon 7 (Ig6)	4	0	2	5	n.a	4	0	1	0	n.a

1 *p* values are according to a two-tailed Fisher's exact test. n.a., not assessed.

The test was performed on raw frequencies of alleles as well on frequencies corrected for minor allele frequency effects (MAF). This correction was done by eliminating all allele frequencies lower than 0.15 when considering all *Dr. melanogaster* populations.

### Contrasting patterns in Epitopes I and II

In *Da. magna* non-synonymous polymorphism was higher in epitope II than in the other regions (Table 3). Likewise non-synonymous divergence is nearly an order of magnitude higher in epitope II compared to epitope I and the remaining exon regions and also compared to exon 10 (Table 3). Contrastingly, synonymous site divergence between *Da. magna* and *Da. lumholtzi* was similar for epitope I, epitope II, and the remaining exon regions of arrays 4 and 6 (Table 3). However, neither the MK-test on epitope II nor the HKA-test comparing epitope II to all remaining regions indicated a significant deviation from neutrality, although there was a tendency for excess non-synonymous polymorphism in epitope II (Table 4). When array 6 was considered alone, this excess of non-synonymous polymorphism was significant (p = 0.04, Table 4), mostly due to exon 6.7 (Fig. S2). This effect disappeared, however, if alleles with a frequency lower than 0.15 were excluded from the analysis (Table 4).

Likewise, in *Dr. melanogaster* array 6 epitope II coding regions exhibited a significant excess of non-synonymous polymorphism relative to the levels of divergence estimated between *Dr. melanogaster* and an inferred ancestral sequence [10]. After removing minor allele frequencies (less than 0.15), the excess of nonsynonymous polymorphism was stronger because mainly synonymous mutations were excluded (Table 5). It is not possible to accurately estimate allele frequencies from the data obtained by [10] in order to know whether the non-synonymous derived alleles are common in the populations analyzed. However, the same derived non-synonymous alleles are present in several of the *Dr. melanogaster* populations surveyed around the world suggesting that they are not rare variants (Table S3).

### Testing for positive selection in epitope II regions in *Drosophila*

The ML analysis implemented in PAML and HYPHY did not yield significant evidence for positive selection in arrays 4 and 6 in the *melanogaster* group, when the entire orthologous coding regions of the two arrays were analyzed, (Table 6, HYPHY results not shown). When the dN/dS of epitope II coding regions was contrasted with the remaining exon regions for both arrays of exons 4 and 6 (Table 6), a model that estimated dN/dS separately for epitope II and for the remaining regions fitted the data better than a model that considered dN/dS to be constant throughout the entire exons. The dN/dS estimates of epitope II coding regions were significantly higher than for the remaing regions, but not higher than 1 (*p*<0.001 in both cases, Table 6).

**Table 6:** Likelihood ratio tests and maximum likelihood estimates of dN/dS for six *Drosophila* species of the *melanogaster* group.

Gene region (Models tested)	N° variable sites	LRT	Parameter estimates
Array 4 total			
(M1a^1^ vs. M2a^2^)	292	n.s.	ω_0_ = 0.009 (96%)^3^
(M7 vs. M8)			ω_1&2_ = 1 (4%)^3^
Epitopes II	84	χ2 = 52^4^;df = 1;	dN/dS = 0.11
Remaining	208	p<0.001	dN/dS = 0.006
Array 6 total			
(M1a^1^ vs. M2a^2^)	784	n.s.	ω_0_ = 0.03 (94%) 3
(M7 vs. M8)			ω_1&2_ = 1 (6%) 3
Epitopes II	242	χ2 = 119^4^;df = 1;	dN/dS = 0.19
Remaining	542	p<0.001	dN/dS = 0.03

Abbreviation: LRT, Likelihood ratio test.

1 M1a: ω_0_ varies between 0 and 1 whereas ω_1_ = 1;

2 M2a adds to M1a, ω_2_>1, which is estimated from the data;

3 proportions of sites under ω_0_, ω_1_, and ω_2_.

4 Tests whether the dN/dS relative to the two partitions are significantly different from each other.

### Divergence between paralogues

The selective constrains acting before and after the duplications of exons 6 differed according to our branch model analysis (Table S4, p<0.001). The average ω over all sites and branches leading to paralogous exons was 0.26 whereas the branches leading to orthologous exons had average ω of 0.094. The branch site analysis on several branches did not provide evidence for a role of positive selection in the divergence between the paralogues (Table S4).

## Discussion

### Insights into exons duplications in arrays 4 and 6

The duplicated exons of arrays 4 and 6 contribute to Dscam isoform diversity due to alternative splicing [11]. Selection on duplicated genes occurs at two levels: on copy numbers and on new mutations within the duplicated forms [39]. In *Daphnia*, we did not find any copy number polymorphism in array 4 among closely related species. This is consistent with results from insects, which indicate that the structure of array 4 is ancient and remained relatively unchanged throughout the evolutionary history of insects [40]. In contrast, the number of exons in array 6 is larger than in array 4 [40] (this study). The reasons for these differences are unknown and our results do not allow distinguishing whether constraints or adaptive evolution might explain them.

Much of the sequence diversification of paralogous exons in arrays 4 and 6 seems to have predated the most recent speciation events, and, in both arrays, exons do not seem to have undergone much concerted evolution, but rather evolved under a birth-and-death evolution process [3]. This is supported by the apparent absence of recent gene conversion events, which is surprising as gene conversion occurs in the majority of other multi-copy gene families [33]. Likely there is selection against gene conversion because it would homogenize exon sequences, thus diminishing the repertoire of different Dscam isoforms. Functional studies showed that Dscam isoform diversity is indeed necessary for the correct development of the nervous system [5]. Interestingly, other important multi-copy immunity related gene families, such as MHC, immunoglobulins, and T-cell receptors, evolve also mainly by birth-and-death evolution rather than by concerted evolution [33].

### Polymorphism and divergence in arrays 4 and 6

Standard tests did not provide evidence for positive selection in arrays 4 and 6 as a whole in *Da. magna*. Rather, all three studied regions showed a tendency for an excess of non-synonymous polymorphism (significant only for array 4). While this can be interpreted as an indication of balancing selection, most of the non-synonymous polymorphisms segregate at low frequency, so that they may also represent segregating, slightly deleterious variants [38]. Also in *Dr. melanogaster*, the excess of non-synonymous polymorphisms in arrays 4 and 6 is mainly caused by low frequency variants. This might derive from the action of purifying selection on the alternatively spliced exons being weaker than on constitutively expressed exons because the former are less expressed than the latter. Yet, rare alleles may also be maintained by time-delayed negative frequency dependent selection which has been described for host-parasite systems [41], [42]. Under this kind of selection, there is a time lag between the allele frequencies and the selection acting on the allele, so that (in contrast to e.g., overdominant selection), allele frequencies are expected to fluctuate in different populations and alleles can be rare for a considerable amount of time [41], [42]. Furtermore, sporadic fixation of alleles may occur and low synonymous variation is predicted due to bottlenecks for the different alleles [43]. Consistent with this prediction, in *Da. magna*, array 4 exons have low synonymous variation. However, in contrast *Dr. melanogaster* tends to have high synonymous variation across the entire *Dscam* gene (Tab. 3).

### The evolution of epitopes I and II

Structural data suggest that epitope I is a crucial unit engaged in the formation of Dscam homologous dimers between the surface of neurons, whereas epitope II is oriented towards the outside of the Dscam protein and is a putative antigen binding region [9]. Within species, the paralogous exon regions of arrays 4 and 6 coding for epitopes I and II have diverged more than the remaining regions of the gene (Table 3). In contrast, divergence between orthologous exon regions coding for epitopes I is much lower than between orthologous exon regions coding for epitopes II in both *Daphnia* (this study) and *Drosophila* [9]. These patterns suggest that the divergence between paralogs is ancient. Intriguingly, however, epitopes I do not seem to have evolved much since then, except by exon duplications, whereas epitopes II have continued to accumulate differences, which is seen in the increased divergence of orthologous sequence between closely related species (Table 3).

### Potential balancing selection in epitopes II

While much of the sequence divergence between paralogous exons may be ancient, allowing high isoform diversity, divergence driven by selection may still be ongoing in some parts of the gene, particularly if any parts of the gene are involved in ongoing coevolution with parasites. Epitope II coding regions of exons 6 in both *Daphnia* and *Drosophila*, show an excess of nonsynonymous polymorphisms relative to the divergence levels. In *Dr. melanogaster*, this effect is still visible after excluding low frequency alleles and may thus suggest balancing selection [44]. In *Dr. melanogaster* allele frequencies could not be inferred with great accuracy, but we found that the same derived non-synonymous alelles segregate in the several *Dr. melanogaster* populations around the world, which suggests that these alleles are not slightly deleterious and are not artifacts due to PCR or sequencing errors (Table S3). Additionally, some of these alleles are present in other distantly related *Drosophila* species, raising the possibility that some of those could be trans-specific polymorphisms (Table S3). However, we did not find high levels of non-synonymous nucleotide polymorphism in Epitope II coding regions, in contrast to that found in the resistance genes *APL1* and *TEP1* of *Anopheles gambiae* to *Plasmodium falciparum*, whose very high levels of non-synonymous polymorphism are presumably a result of balancing selection and gene conversion [45], [46].

If balancing selection is maintained for a long time, it is expected to lead to strong linkage disequilibrium (LD) and to elevated neutral variation at linked sites [44], [47]. In *Da. magna* the synonymous site diversity of exon 6.7 is among the highest of all sampled exons in array 6 (π_s_ = 0.012), but synonymous site diversity of the whole array 6 is only slightly higher than that of the constitutive exon 10. In addition, we did not find elevated LD in the region (results not shown). Thus if any balancing selection acts on the region, it is unlikely to be long-term balancing selection, as found in some other immunity genes such as MHC [48]. In the *Dr. melanogaster* populations, Dscam synonymous diversity tends to be high across the whole gene (Table S2), but it is not possible to estimate whether there are any sites in LD with epitope II coding sites given that no haplotype information is available.

An alternative explanation, as discussed above, is that epitopes II are under negative frequency dependent selection. In such case, due to periodic bottlenecks, non-synonymous diversity is not expected to be elevated [43] and the prediction for LD is less clear. However, to differentiate between overdominant and negative frequency dependent selection acting on this region would require better estimates of allele frequencies among different populations both in *Daphnia* and *Drosophila*. In summary, our data do not currently allow us to distinguish between the hypothesis of negative frequency-dependent selection and the hypothesis of relaxed selective constraints, although the fact that the same derived alleles segregate in several *Drosophila* populations suggest a likely action of some form of balancing selection.

Maximum likelihood codon based site models have been shown to be powerful at detecting balancing selection in MHC [28], [49]. Yet many of the studies on MHC involved comparison of paralogous MHC alleles [48], [50] [28], [49]. In Dscam, paralogous exons diverged too extensively (array of exons 6 tree length for dS is 104.4 in *Dr. melanogaster*) to be included in a reliable site model analysis [51]. The site model analysis of orthologous exons of arrays 4 and 6 in six *Drosophila* species revealed that although epitopes II evolve faster than the remaining regions of these arrays, there is no evidence that this is driven by positive selection. However, as discussed in the supplementary section (Table S2), our analysis has most likely low power for detecting balancing selection.

### Involvement of epitope II in immune recognition in insects and crustaceans

Despite some differences, the results obtained with *Daphnia* and *Drosophila* point to similar molecular patterns of Dscam. The gene does not have high nucleotide diversity in both *Da. magna* and *Dr. melanogaster*. Instead, Dscam diversity is generated by alternative splicing of duplicated exons (more than 13000 and 30000 protein isoforms can potentially be expressed in *Da. magna* and *Dr. melanogaster*, respectively) and there is selection to preserve the diversity caused by duplication and divergence. In both taxa, epitope II coding regions diverged more than the rest of the gene, but in *Drosophila* we could not show that this high substitution rate was due to adaptive evolution. Epitope II coding regions harbor an excess of non-synonymous polymorphism in relation to the divergence levels observed. This could be maintained by balancing selection but also be influenced by segregating slightly deleterious mutations as discussed previously, which would suggest lower constraints on this part of the Dscam molecule.

Nevertheless, some of the segregating epitope II amino acids in both *Da. magna* and *Dr. melanogaster* populations might considerably change the binding capacities of the epitope (Fig. 2). In *Da. magna* arginine and glycine (exon 6.7) and in *Dr. melanogaster* arginine and methionine (exon 6.24) or asparagine and lysine (exon 6.39). In the case of the arginine polymorphism, the amino acid variants have exactly the same position in the epitope in both taxa in non-orthologous exons (Fig. 2). Furthermore, at this position glycine is a hallmark amino acid of many Ig domains [52] which corroborates the idea that this polymorphism might not be neutral. In *Da. magna* the arginine/glycine polymorphism showed an intermediate-frequency polymorphism with 54% of the analyzed individuals being homozygous for glycine, 30% being homozygous for arginine, and 17% being heterozygous across different populations. Both *Da. lumholtzi* and *Da. pulex* have glycine at this site.

Epitopes II are formed by the interception of two interstrand loops belonging to Ig2 and Ig3 domains (Fig. 2). This resembles “complementary determining regions” of T cell receptors or antibodies of the Immunoglobulin superfamily that, respectively, bind peptides or native antigenic determinants from pathogens (Fig. 2). A similar epitope in hemolin, a molecule involved in immunity in leptidopterans, has been suggested to harbor a similar region involved in bacterial lipopolysaccharide binding [53]. These and other structural similarities constitute circumstantial evidence for an involvement of Dscam in immunity, yet the molecular patterns we have found are not unequivocal.

Genes of the immune system involved in recognition, such as MHC, present hallmarks of long-term balancing selection; elevated levels of synonymous diversity and deeply diverged, trans-specific alleles. However, such strong patterns are not found in Dscam. It remains a challenge in the field of arthropod immunology to uncover the underlying mechanisms of the Dscam function. Expression by effector cells of the immune system such as hemocytes, is not in itself a guarantee of an involvement in immune recognition. Dscam diversity could play there a role similar to that played in neurons, controlling interactions between hemocytes inside the body.

## Supporting Information

Figure S1 **Array 4 (A) and array 6 (B) partitions of epitope I and epitope II in Da. magna.** Polymorphic positions are indicated by amino acids with the size of the letter being proportional to the frequencies of each amino acid. The colors represent the chemical properties of amino acids: polar (green), basic (blue), acidic (red) and hydrophobic (black). This figure was created with WebLogo (http://weblogo.berkeley.edu/logo.cgi).
(DOC)

Figure S2 **Sliding window analysis across array 6 exons of the ratios of nonsynonymous nucleotide diversity πa to synonymous nucleotide diversity πs in Da. magna and of nonsynonymous divergence Ka to synonymous divergence Ks ratio between D. magna and D. lumholtzi.** The sliding window analysis was done with DNAsp using a 50 bp window length with a 10 bp step size. The intron/exon boundaries as well as the locations of epitopes I (white bars, black dots) and epitopes II (grey bars) are indicated below the x-axis.
(DOC)

Figure S3 A) Maximum likelihood tree of array 6 exons in the melanogaster subgroup including orthologous and paralogous exons. Support values at nodes are bootstrap values (100 bootstrap replicates). Branch length estimates the expected number of nucleotide substitutions per codon using the one-ratio model, and the tree topology and branch lengths were used to fit different models. The tree is rooted for convenience at the midpoint but all analyses were done with an unrooted topology. Red branches with arrows indicate branches for which the presence of aminoacid sites that evolved with ω>1 was tested using branch-site models implemented in PAML [31], [32]. The branches chosen were the ones leading to duplicated exons where we detected an excess of non-synonymous polymorphism in Dr. melanogaster using McDonald-Kreitman tests. the PAML tests used smaller subtrees (grey boxes). B) Schematic representation of branch models. We used these models to test whether selection changed after duplication, that is whether orthologous and paralogous branches differ in ω (model R2). The null model R1 assumes that all branches in the tree have the same ω.
(DOC)

Table S1 **Non-synonymous polymorphisms and non-synonymous divergence in the duplicated exons of Dscam in Daphnia.** ^a^ Array and exon numbering as in [3]. ^b^ Codon numbering within each exon. (II) indicates that the codon is in epitope II. i and ii refer respectively to nucleotides 658 and 659 in the same codon. ^c^ P indicates a polymorphism within Da. magna, D a fixed difference between Da. magna and Da. lumholtzi, and P/D a polymorphic site within Da. magna at which Da. lumholtzi has a third amino acid. ^d^ The first amino acid corresponds to the more common allele in the case of polymorphic (P and P/D sites). The last amino acid designates the one present in Da. lumholtzi (D and P/D sites). ^e^ Frequency of the most common allele.
(DOC)

Table S2 **Random sites model** [**23**] **likelihood ratio tests (LRT) for positive selection at MHC Class I locus B in six primate species.** One allele per species was randomly chosen from Genebank (HQ231327.1 Homo sapiens, DQ026306.1 Gorilla gorilla, CR860073.1 Pongo abelii, AAB08074.1 Hylobates lar, AAY59437.1 Pan troglodytes, AAA50178.1 Pan paniscus). This analysis was done to assess the power of the random site model tests in our analysis of the Drosophila data, According to the results, the amino acid variation observed between the orthologous MHC alleles was more likely explained by neutral evolution (i.e., no significant signs of positive selection were found), which suggests that our site model analysis is not very powerful at detecting diversifying selection. ^a^ ω0, ω1, ω2 indicate the estimated values of ω under the conditions of each model; M1a: 0<ω0<1, ω1 = 1; M2a adds to M1a ω2>1, which is estimated from the data; within brackets is the proportion of sites estimated to be in each category of ω. In M7, 0≤ω≤1 and p and q are parameters of the beta distribution. M8 adds one extra class of sites ω≥1 to M7.
(DOC)

Table S3 **Non-synonymous polymorphisms in epitope II regions of array 6 exons in Dr. melanogaster.** Shown are only polymorphisms at which the overall frequency of the rarer allele exceeds 0.15.The amino acids present at the orthologous codons in other Drosophila species is shown as well. ^a^ Polymorphism data and codon numbering from [10]. n.o. indicates that no orthologous exon was found in this species.
(DOC)

Table S4 **Branch models and branch-site models applied to the exons of array in the melanogaster subgroup.** Likelihood ratio test (LRT), parameter estimates (ω), and positively selected sites are shown. In branch-site models the branch of interest is called foreground branch (Fig. S3, red branches with arrows) and all the other branches in the tree are called background branches. ^a^ Parameter estimates under the alternative models: ω0:dN/dS<1; ω1: dN/dS = 1, ω2aF = dN/dS>1 (alternative hypothesis) or dN/dS = 1 (null hypothesis) on the foreground branch and dN/dS<1 on background branches,ω2aB; ω2bF = dN/dS>1 (alternative hypothesis) or dN/dS = 1 (null hypothesis) on the foreground branch and dN/dS = 1 on background branches. ^b^ Sites inferred to be under positive selection at the 95% (*) or 99% (**) by Bayes Empirical Bayes analysis.
(DOC)

Table S5 **Estimates of divergence between Da. magna and Da. similis, as well as McDonald Kreitman tests for the comparison between the two species.** No polymorphisms were excluded for this analysis. ^a^ p values are according to a two-tailed Fisher's exact test.
(DOC)
