# Supplementary figures and images for: Population Genetics of Duplicated Alternatively Spliced Exons of the Dscam Gene in Daphnia and Drosophila
Source: PLoS One. 2011 Dec 12;6(12):e27947. doi: 10.1371/journal.pone.0027947 (PMC3236188; doi:10.1371/journal.pone.0027947)

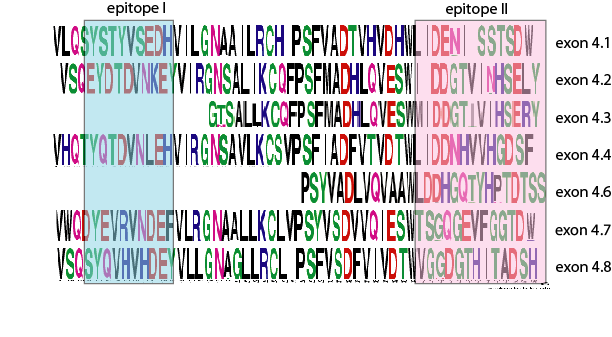


A)

B)


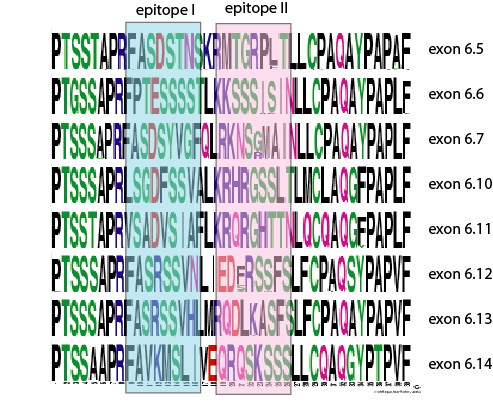

Supplement: Figure S1 — Array 4 (A) and array 6 (B) partitions of epitope I and epitope II in Da. magna. Polymorphic positions are indicated by amino acids with the size of the letter being proportional to the frequencies of each amino acid. The colors represent the chemical properties of amino acids: polar (green), basic (blue), acidic (red) and hydrophobic (black). This figure was created with WebLogo (http://weblogo.berkeley.edu/logo.cgi). (DOC) [file pone.0027947.s001.doc]

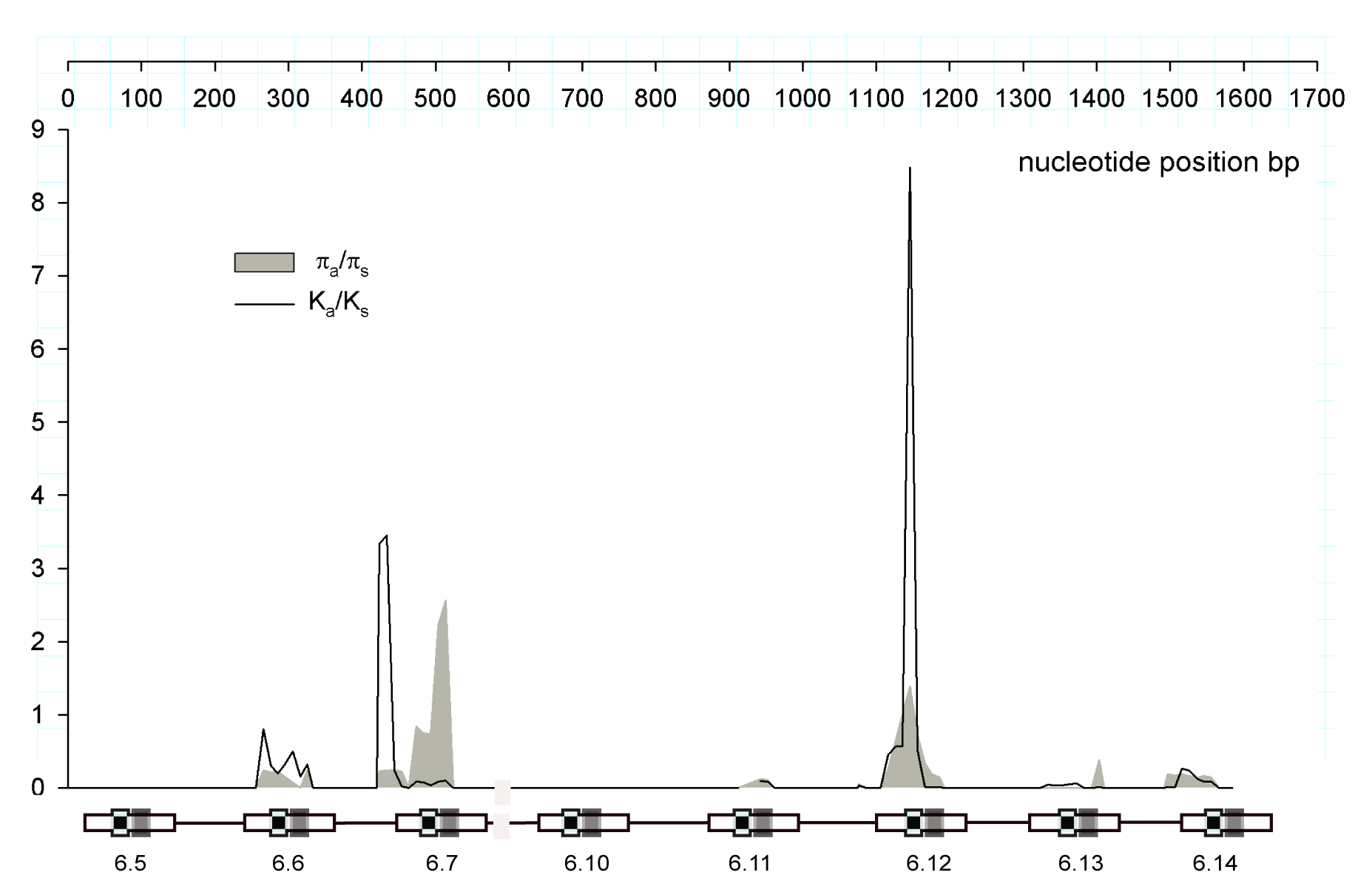

Supplement: Figure S2 — Sliding window analysis across array 6 exons of the ratios of nonsynonymous nucleotide diversity πa to synonymous nucleotide diversity πs in Da. magna and of nonsynonymous divergence Ka to synonymous divergence Ks ratio between D. magna and D. lumholtzi. The sliding window analysis was done with DNAsp using a 50 bp window length with a 10 bp step size. The intron/exon boundaries as well as the locations of epitopes I (white bars, black dots) and epitopes II (grey bars) are indicated below the x-axis. (DOC) [file pone.0027947.s002.doc]
