# Supplementary material for: Population Genetics of Duplicated Alternatively Spliced Exons of the Dscam Gene in Daphnia and Drosophila
Source: PLoS One. 2011 Dec 12;6(12):e27947. doi: 10.1371/journal.pone.0027947 (PMC3236188; doi:10.1371/journal.pone.0027947)
Supplement: Figure S3 — A) Maximum likelihood tree of array 6 exons in the melanogaster subgroup including orthologous and paralogous exons. Support values at nodes are bootstrap values (100 bootstrap replicates). Branch length estimates the expected number of nucleotide substitutions per codon using the one-ratio model, and the tree topology and branch lengths were used to fit different models. The tree is rooted for convenience at the midpoint but all analyses were done with an unrooted topology. Red branches with arrows indicate branches for which the presence of aminoacid sites that evolved with ω>1 was tested using branch-site models implemented in PAML [31], [32]. The branches chosen were the ones leading to duplicated exons where we detected an excess of non-synonymous polymorphism in Dr. melanogaster using McDonald-Kreitman tests. the PAML tests used smaller subtrees (grey boxes). B) Schematic representation of branch models. We used these models to test whether selection changed after duplication, that is whether orthologous and paralogous branches differ in ω (model R2). The null model R1 assumes that all branches in the tree have the same ω. (DOC) [file pone.0027947.s003.doc]

|  | | Codon^a^ | | | | | | |
| --- | --- | --- | --- | --- | --- | --- | --- | --- |
| Species | Population | 65 | 9502 | 1027 | 1109 | 1547 | 1598 | 1625 |
| *Dr. melanogaster* | Athens | S/G | R | P/L | A/S | N/K | I/S | A/V |
|  | Florida | S/G | R | P/L | A/S | N/K | I/S | A/V |
|  | French Polynesia | S/G | R | P/L | A/S | N | S | A/V |
|  | Gabon | S/G | R/M | P | A | N/K | I/S | A |
|  | Japan | S/G | R/M | P/L | A/S | N/K | I/S | A/V |
|  | Kenya | S/G | R | P/L | A/S | N/K | I/S | A/V |
| Ancestral | | G | R | P | A | N | S | A |
| *Dr. simulans* | | G | R | A | A | K | S | A |
| *Dr. sechellia* | | G | R | P | A | K | S | A |
| *Dr. yacuba* | | G | R | A | A | K | S | n.o. |
| *Dr. erecta* | | G | R | S | A | K | I | A |
| *Dr. ananassae* | | G | Q | S | S | N | S | A |
| *Dr. pseudoobscura* | | G | R | Q | S | N | S | A |
| *Dr. persimilis* | | G | R | Q | S | N | S | A |
| *Dr. willistoni* | | Q | n.o. | Q | A | N | n.o. | D |
| *Dr. mojavensis* | | E | R | Q | G | N | S | A |
| *Dr. virilis* | | E | Q | Q | T | N | N | G |
| *Dr. grimshawi* | | K | Q | Q | A | N | S | P |
