# Supplementary material for: Population Genetics of Duplicated Alternatively Spliced Exons of the Dscam Gene in Daphnia and Drosophila
Source: PLoS One. 2011 Dec 12;6(12):e27947. doi: 10.1371/journal.pone.0027947 (PMC3236188; doi:10.1371/journal.pone.0027947)
Supplement: Table S1 — Non-synonymous polymorphisms and non-synonymous divergence in the duplicated exons of Dscam in Daphnia. a Array and exon numbering as in [3]. b Codon numbering within each exon. (II) indicates that the codon is in epitope II. i and ii refer respectively to nucleotides 658 and 659 in the same codon. c P indicates a polymorphism within Da. magna, D a fixed difference between Da. magna and Da. lumholtzi, and P/D a polymorphic site within Da. magna at which Da. lumholtzi has a third amino acid. d The first amino acid corresponds to the more common allele in the case of polymorphic (P and P/D sites). The last amino acid designates the one present in Da. lumholtzi (D and P/D sites). e Frequency of the most common allele. (DOC) [file pone.0027947.s004.doc]

| Exon^a^ | Codon ^b^ | State^c^ | AA^d^ | Frequency (%)^e^ |
| --- | --- | --- | --- | --- |
| 4.1 | 19 | P | A/T | 96.4 |
| 4.1 | 44 (II) | D | N/S |  |
| 4.2 | 90 | P | E/D | 96.4 |
| 4.2 | 100 (II) | D | N/T |  |
| 4.3 | 107 | P | T/N | 92.80 |
| 4.3 | 111 | D | L/I |  |
| 4.3 | 135 (II) | D | I/T |  |
| 4.6 | 211 (II) | P | D/A | 96.4 |
| 4.6 | 215 (II) | D | T/S |  |
| 4.6 | 218 (II) | D | P/Q |  |
| 4.7 | 243 | P | A/V | 96.4 |
| 4.7 | 264 (II) | D | G/S |  |
| 4.7 | 275 (II) | P | T/R | 92.80 |
| 4.8 | 294 | D | A/T |  |
| 4.8 | 317 (II) | D | G/D |  |
| 6.6 | 38 | D | F/N |  |
| 6.6 | 39 | D | F/N |  |
| 6.6 | 62 (II) | D | I/A |  |
| 6.6 | 63 | P | S/F | 93.75 |
| 6.6 | 78 | D | F/Y |  |
| 6.7 | 84 | P | A/S | 93.75 |
| 6.7 | 102 (II) | P | G/R | 71.8 |
| 6.7 | 103 (II) | P | M/I | 93.75 |
| 6.1 | 75 | P | F/Y | 87.5 |
| 6.12 | 81 | P | P/S | 93.75 |
| 6.12 | 101^i^ (II) | D | F/S/T |  |
| 6.12 | 101^ii^ (II) | P/D | F/S/T | 75 |
| 6.12 | 104 (II) | P | S/N | 93.75 |
| 6.12 | 111 | P | A/T | 93.75 |
| 6.13 | 142 (II) | D | K/T |  |
| 6.14 | 164 | P | A/V | 96.87 |
| 6.14 | 176 | P | V/L | 96.87 |
