# Supplementary material for: Population Genetics of Duplicated Alternatively Spliced Exons of the Dscam Gene in Daphnia and Drosophila
Source: PLoS One. 2011 Dec 12;6(12):e27947. doi: 10.1371/journal.pone.0027947 (PMC3236188; doi:10.1371/journal.pone.0027947)
Supplement: Table S2 — Random sites model [23] likelihood ratio tests (LRT) for positive selection at MHC Class I locus B in six primate species. One allele per species was randomly chosen from Genebank (HQ231327.1 Homo sapiens, DQ026306.1 Gorilla gorilla, CR860073.1 Pongo abelii, AAB08074.1 Hylobates lar, AAY59437.1 Pan troglodytes, AAA50178.1 Pan paniscus). This analysis was done to assess the power of the random site model tests in our analysis of the Drosophila data, According to the results, the amino acid variation observed between the orthologous MHC alleles was more likely explained by neutral evolution (i.e., no significant signs of positive selection were found), which suggests that our site model analysis is not very powerful at detecting diversifying selection. a ω0, ω1, ω2 indicate the estimated values of ω under the conditions of each model; M1a: 0<ω0<1, ω1 = 1; M2a adds to M1a ω2>1, which is estimated from the data; within brackets is the proportion of sites estimated to be in each category of ω. In M7, 0≤ω≤1 and p and q are parameters of the beta distribution. M8 adds one extra class of sites ω≥1 to M7. (DOC) [file pone.0027947.s005.doc]

| Model | LRT | Parameters ^a^ |
| --- | --- | --- |
| M1a  vs. M2a | χ2=3.06  df =2 *p*=0.2 | M1a*: ω_0_*=0 (71%) *ω_1_*=1 (29%)  M2a: *ω_2_*=2 (21%) |
| M7  vs.  M8 | χ2=3.1  df=2  *p*=0.2 | M7: *p*=0.005; *q*=0.011  M8: *‘p* =4.66, *‘q*=88 *ω*=2 (20%) |
