# Supplementary material for: Population Genetics of Duplicated Alternatively Spliced Exons of the Dscam Gene in Daphnia and Drosophila
Source: PLoS One. 2011 Dec 12;6(12):e27947. doi: 10.1371/journal.pone.0027947 (PMC3236188; doi:10.1371/journal.pone.0027947)
Supplement: Table S4 — Branch models and branch-site models applied to the exons of array in the melanogaster subgroup. Likelihood ratio test (LRT), parameter estimates (ω), and positively selected sites are shown. In branch-site models the branch of interest is called foreground branch (Fig. S3, red branches with arrows) and all the other branches in the tree are called background branches. a Parameter estimates under the alternative models: ω0:dN/dS<1; ω1: dN/dS = 1, ω2aF = dN/dS>1 (alternative hypothesis) or dN/dS = 1 (null hypothesis) on the foreground branch and dN/dS<1 on background branches,ω2aB; ω2bF = dN/dS>1 (alternative hypothesis) or dN/dS = 1 (null hypothesis) on the foreground branch and dN/dS = 1 on background branches. b Sites inferred to be under positive selection at the 95% (*) or 99% (**) by Bayes Empirical Bayes analysis. (DOC) [file pone.0027947.s007.doc]

| Models | LRT | *Parameters* | Positively selected sites^b^ |
| --- | --- | --- | --- |
| *Branch models* |  |  |  |
| One-ratio (R1)  vs.  Two-ratios (R2) | χ2=46  df =1 *p*<0.001 | *ω_1_*=0.26 *ω_2_*=0.094 |  |
| *Branch-site models* |  | *Parameters^a^* |  |
| Foreground branch (**a**)  vs.  Background | χ2=1.46  df =1 *p*=0.2 | *ω_0_*=0.07 *ω_1_*=1 *ω_2a_*B=0.07 *ω_2a_*F=5.43 *ω_2b_*F=5.43 | 10T**; 15 S*; 16 R*; 25 S** |
| Foreground branch (**b**)  vs.  Background | χ2=0.38  df =1 *p*=0.55 | *ω_0_*=0.08 *ω_1_*=1 *ω_2a_*B =0.08 *ω_2a_*F=2.32 *ω_2b_*F=2.32 | 18 T*; 21 P**; 37 V** |
| Foreground branch (**c**)  vs.  Background | χ2=0.09  df =1  *p*=0.8 | *ω_0_*=0.08*ω_1_*=1 *ω_2a_*B =0.08 *ω_2a_*F =1 *ω_2b_*F=1 |  |
| Foreground branch (**d**)  vs.  Background | χ2=0  df =1  *p*=1 | *ω_0_*=0.02 *ω_1_*=1 *ω_2a_*B =0.02 *ω_2a_*F =1 *ω_2b_*F=1 |  |
| Foreground branch (**e**)  vs.  Background | χ2=0  df =1  *p*=1 | *ω_0_*=0.05 *ω_1_*=1 *ω_2a_*B =0.05 *ω_2a_*F =1 *ω_2b_*F=1 |  |
| Foreground branch (**f**)  vs.  Background | χ2=0  df =1  *p*=1 | *ω_0_*=0.08 *ω_1_*=1 *ω_2a_*B =0.08 *ω_2a_*F =1 *ω_2b_*F=1 |  |
