# Supplementary material for: Population Genetics of Duplicated Alternatively Spliced Exons of the Dscam Gene in Daphnia and Drosophila
Source: PLoS One. 2011 Dec 12;6(12):e27947. doi: 10.1371/journal.pone.0027947 (PMC3236188; doi:10.1371/journal.pone.0027947)
Supplement: Table S5 — Estimates of divergence between Da. magna and Da. similis, as well as McDonald Kreitman tests for the comparison between the two species. No polymorphisms were excluded for this analysis. a p values are according to a two-tailed Fisher's exact test. (DOC) [file pone.0027947.s008.doc]

| Gene region | *Da. magna vs Da. similis* | | | | | | | |
| --- | --- | --- | --- | --- | --- | --- | --- | --- |
|  | Divergence (k) | | | Fixed | | Polymorphic | | *p*^a^ |
|  | Ks | Ka | Ka/Ks | Syn | Nonsyn | Syn | Nonsyn |  |
| Array 4 Total | 0.094 | 0.011 | 0.117 | 21 | 8 | 4 | 6 | 0.12 |
| Epitopes II | 0.07 | 0.027 | 0.35 | 5 | 6 | 2 | 2 | 1 |
